# On Pruritus Vulvæ and Diabetes

**Published:** 1878-08

**Authors:** Alfred Wiltshire

**Affiliations:** M. D., M. R. C. P. Lond., Wimpole-Street, W.


### On Pruritus Vulvae and Diabetes.

By ALFRED WILTSHIRE, M. D., M. R. C. P. Lond.,

A FEW years ago, at a meeting of the Harveian Society, I called attention to the frequent association of pruritus vulvæ with diabetes. As continued observation confirms and strengthens the statements then made, and as my observations have never been made known otherwise than as above stated and in my lectures, I desire again very briefly to call attention to and emphasize the fact that pruritus vulvæ is often the only sympton of diabetes, and to point out the desirability of a systematic examination of the urine for sugar in suspicious cases.

Diabetes is only one among many affections with which pruritus vulvæ may be associated ; but notwithstanding that it is mentioned in some books as a cause of vulvar itching, it is nowhere, that I am aware of, stated with adequate prominence.

Excepting itching, there may be no sympton whatever of diabetes — neither polyuria, loss of flesh, nor large appetite ; and it is not therefore a matter for surprise that the underlying diabetic affection frequently remains unsuspected.

The observations of friends who have become acquainted with my views are confirmatory of them, and show that there is a more frequent connexion between diabetes and pruritus vulvæ than is generally believed. Accordingly, it seems desirable that further attention should be directed to the fact seeing the gravity of the more important affection, It is hoped that a wider diffusion of the knowledge may prove useful to many.

Though matter of high interest, I am not concerned at present to discuss the pathogeny of diabetes; but regarding it from a purely clinical aspect, it is difficult to avoid the conclusion that there are at least two (if no more) forms of diabetes; or, to put it in another way, that sugar, or some allied body equally capable of reducing copper, on the test being applied in the usual way, may often be found in the urine of stout, florid (gouty) persons, as well as in the lean, wasted people who are looked upon as classical types of the disease.

I have at present under my care at St. Mary's Hospital an excellent illustration of this fact. When the patient, a stout, florid middle-aged woman, first came under my care some months ago, she was tormented with violent itching of the privates. Suspecting diabetes, I had the urine examined, and then, and ever since, it has contained an abundance of sugar. She rarely passes more than a normal amount of urine, looks as hearty as can be, and from the first application of the treatment—a borax lotion—has almost lost the itching. As the object of this short paper is merely to put others on the track of diabetes through the pruritic symptom, I will add no more, but await hopefully the statement of the experience of others.—*London Lancet.*

Wimpole-Street, W.

